# Supplementary material for: Multi-antigen intranasal vaccine protects against challenge with sarbecoviruses and prevents transmission in hamsters
Source: Nat Commun. 2024 Jul 23;15:6193. doi: 10.1038/s41467-024-50133-2 (PMC11266618; doi:10.1038/s41467-024-50133-2)
Supplement: Supplementary file 1 — Supplementary Information [file 41467_2024_50133_MOESM1_ESM.pdf]

SUPPLEMENTARY INFORMATION

Multi-antigen intranasal vaccine protects against challenge with sarbecoviruses and prevents transmission in hamsters

Ankita Leekha[1*], Arash Saeedi[1*], Samiur Rahman Sefat[1], Monish Kumar[1], Melisa Martinez-Paniagua[1], Adrian Damian[1], Rohan Kulkarni[1], Kate Reichel[1], Ali Rezvan[1], Shalaleh Masoumi[2], Xinli Liu[2], Laurence JN Cooper[3], Manu Sebastian[3], Courtney M. Sands[4], Vallabh E. Das[4], Nimesh B. Patel[4], Brett Hurst[5], and Navin Varadarajan[1†]

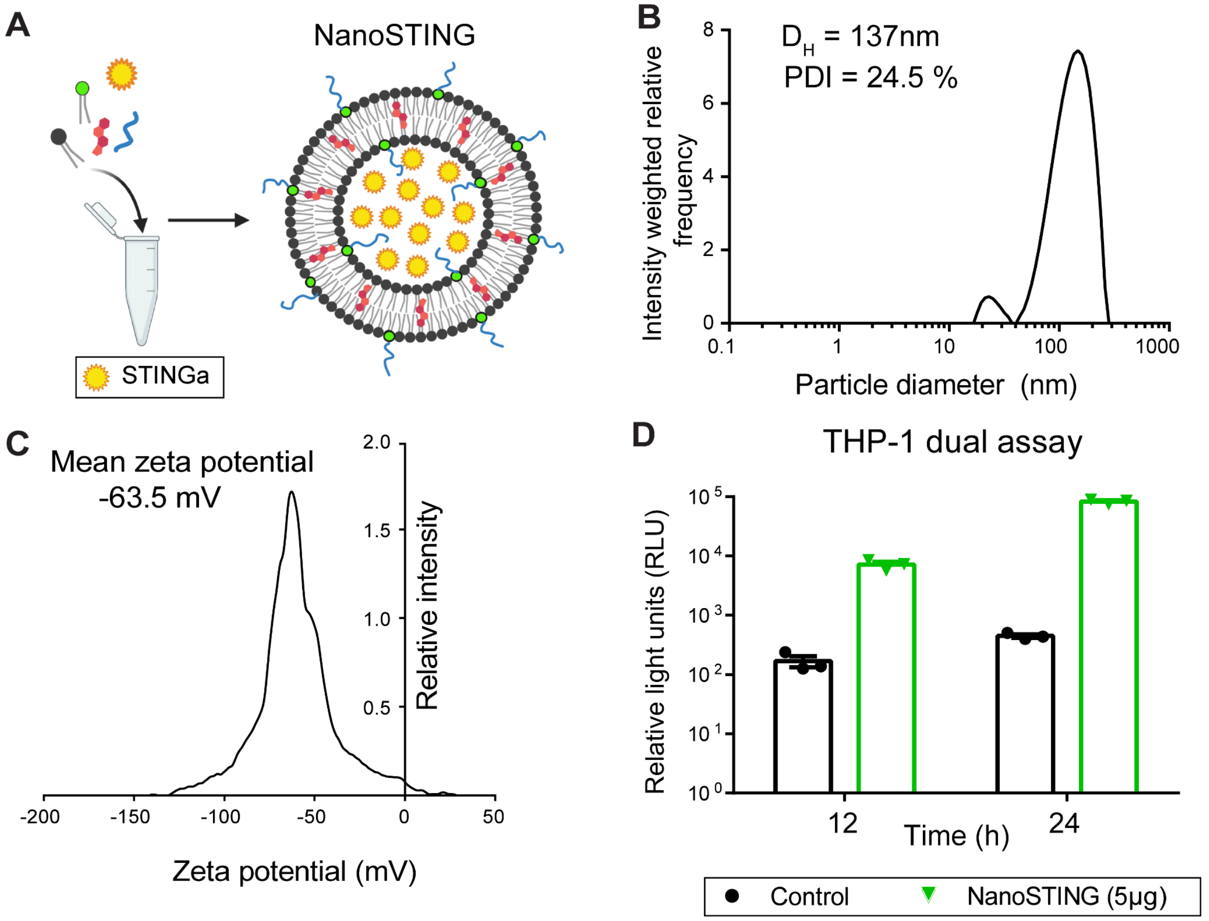

Supplementary Fig. 1: Synthesis and characterization of NanoSTING (related to Figure 1)

(A) Overall schematic of the formulation of NanoSTING. **(B)** Distribution of NanoSTING liposomal particle sizes measured by DLS. **(C)** Zeta potential of the NanoSTING measured by ELS. **(D)** Kinetics of the induction of luciferase in THP1-dual^{TM} cells by NanoSTING (5 μg) at 12h and 24 h. Individual data points represent technical replicates; vertical bars show mean values with error bars representing SEM. Data presented as combined results from one independent experiment. Two-tailed mann-whitney U-test: ****p < 0.0001; ***p < 0.001; **p < 0.01; *p < 0.05; ns: not significant. Supp. Fig. 1A Created with BioRender.com released under a Creative Commons Attribution-NonCommercial-NoDerivs 4.0 International license (https://creativecommons.org/licenses/by-nc-nd/4.0/deed.en). Abbreviations: D_H: hydrodynamic diameter; PDI: polydispersity index, DLS: dynamic light scattering, ELS: electrophoretic light scattering, RLU: relative light units, nm: nanometers, mV: millivolts

Source data are provided as a Source Data file.

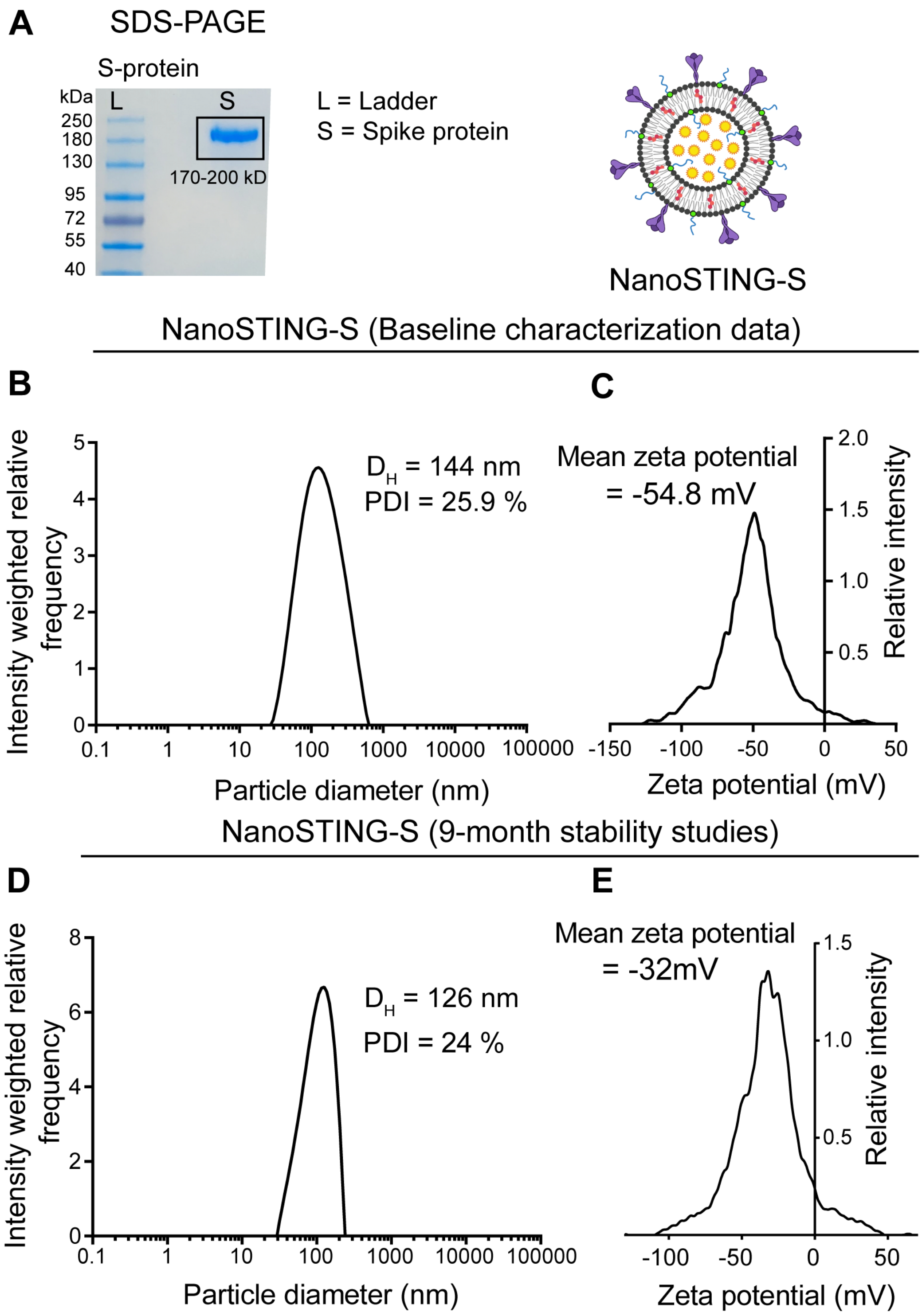

Supplementary Fig. 2: Characterization of NanoSTING-S (related to Figure 1)

(A) Denaturing SDS-PAGE gel of the purified trimeric S protein. **(B)** Distribution of NanoSTING-S liposomal particle sizes measured DLS. **(C)** Zeta potential of the NanoSTING-S measured by ELS. **(D)** Distribution of NanoSTING-S particle sizes measured

by DLS after storage at 4 °C for 9 months. **(E)** Zeta potential of the NanoSTING-S was measured by ELS after storage 4 °C for 9 months. Supp. Fig. 2B Created with BioRender.com released under a Creative Commons Attribution-NonCommercial-NoDerivs 4.0 International license (https://creativecommons.org/licenses/by-nc-nd/4.0/deed.en). Abbreviations: D_H: hydrodynamic diameter; PDI: polydispersity index, DLS: dynamic light scattering, ELS: electrophoretic light scattering, nm: nanometers, mV: millivolts.

Source data are provided as a Source Data file.

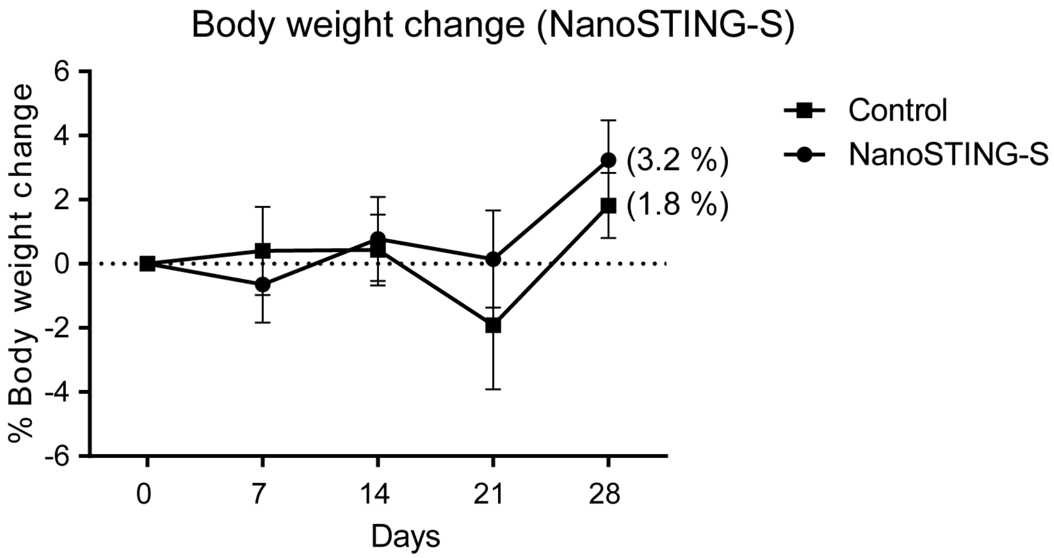

Supplementary Fig. 3: Percentage of bodyweight change of NanoSTING-S vaccinated mice compared to the control mice (related to Figure 1).

Lines depict group mean bodyweight change from day 0; error bars represent SEM. Data presented as combined results from one independent experiment. Gender was not tested as a variable, with only female mice used for the study.

Number of animals used: n=5/group

Source data are provided as a Source Data file.

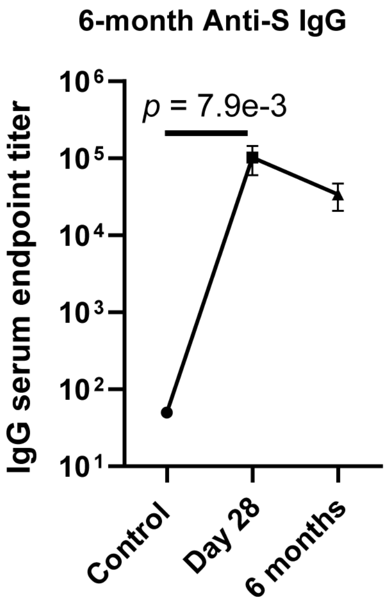

Supplementary Fig. 4: Durability of the serum IgG responses in mice. We immunized the group of 5 *BALB/c* mice with a single dose of NanoSTING-S and evaluated the IgG responses against S protein on day 28 and 6 months after immunization.

Lines depict group mean IgG serum end point titers for indicated time points; error bars represent SEM. Data presented as combined results from one independent experiment. Gender was not tested as a variable, and only female mice were used for the study.

Number of animals used: n=4-5/group

Source data are provided as a Source Data file.

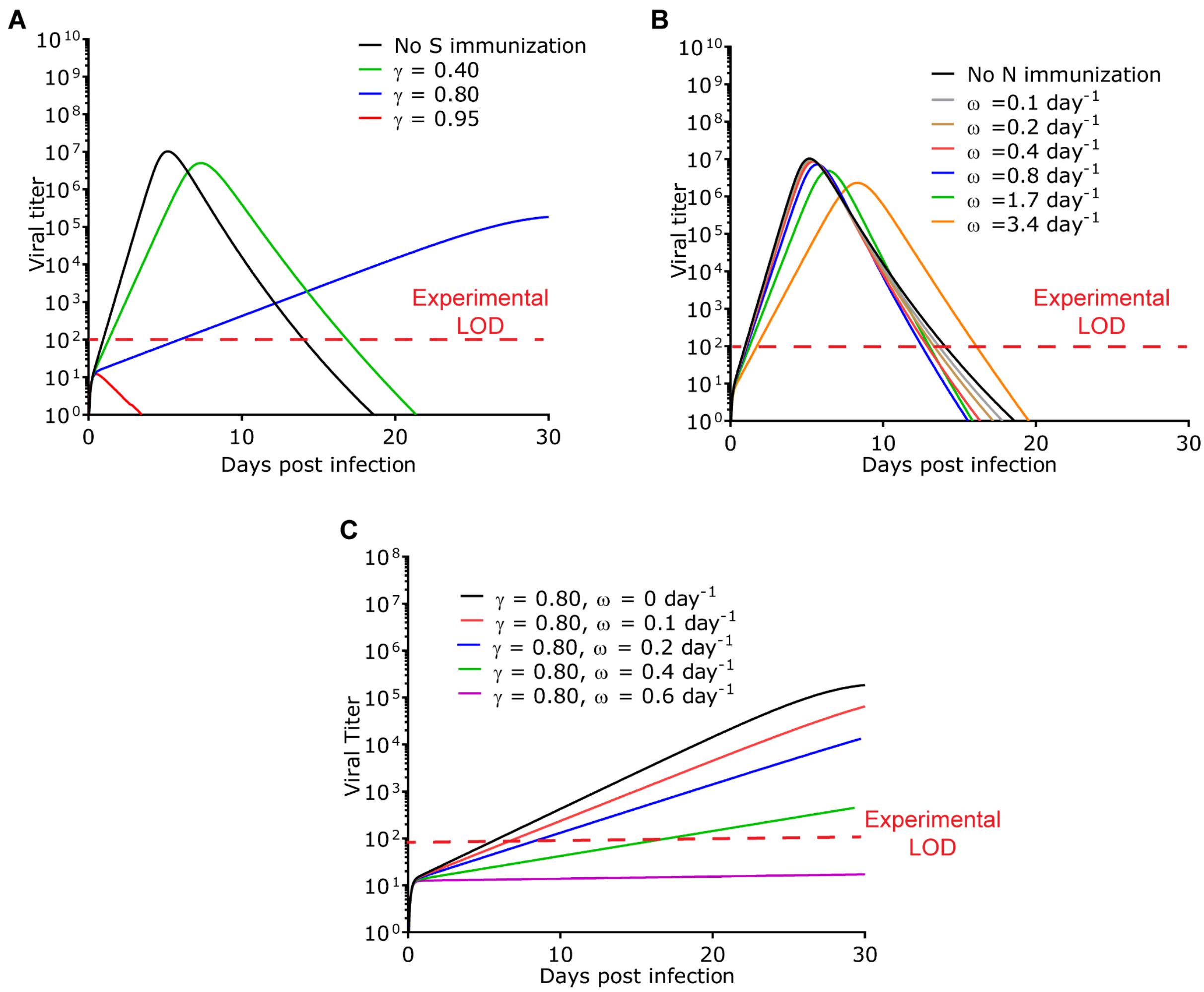

Supplementary Fig. 5: Evolution of viral dynamics with

(A) S immunization (assuming only de-novo blocking of viral entry). Increasing the de-novo blocking efficiency of viral entry decreased the rate of viral infection. Blocking efficiency of 80% (γ = 0.8) significantly reduced the viral titer growth rate. **(B)** N immunization (assuming only cytotoxic T cell killing of infected cells). T-cell responses alone do not reduce the viral load significantly. **(C)** Immunization with both N and S combined. Physiological rates of T cell responses (ω = 0-0.6 day^{-1}) with de-novo blocking of viral entry with 80% efficiency (γ = 0.8) act synergistically to reduce viral replication (related to Figure 2). Dashed red line indicates the experimental limit of detection of viral titers.

Source data are provided as a Source Data file.

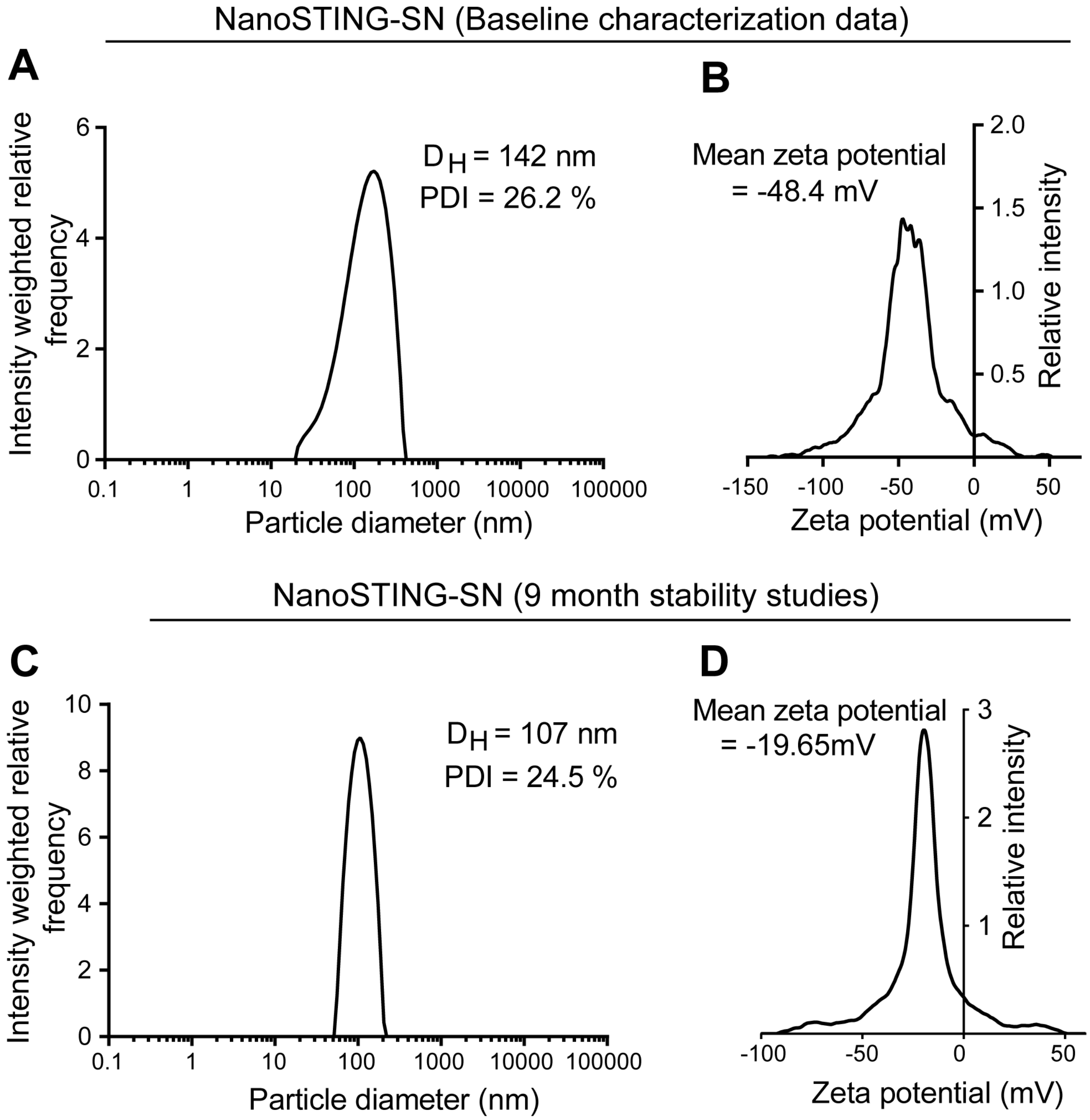

Supplementary Fig. 6: Characterization of NanoSTING-SN (related to Figure 3)

(A) Distribution of NanoSTING-SN particle sizes measured by DLS. **(B)** Zeta potential of the NanoSTING-SN measured by ELS. **(C)** Distribution of NanoSTING-SN particle sizes measured by DLS after storage at 4 °C for 9 months. **(D)** Zeta potential of the NanoSTING-SN was measured by ELS after storage 4 °C for 9 months. Abbreviations: D_H: hydrodynamic diameter; PDI: polydispersity index, DLS: dynamic light scattering, ELS: electrophoretic light scattering, nm: nanometers, mV: millivolts.

Source data are provided as a Source Data file.

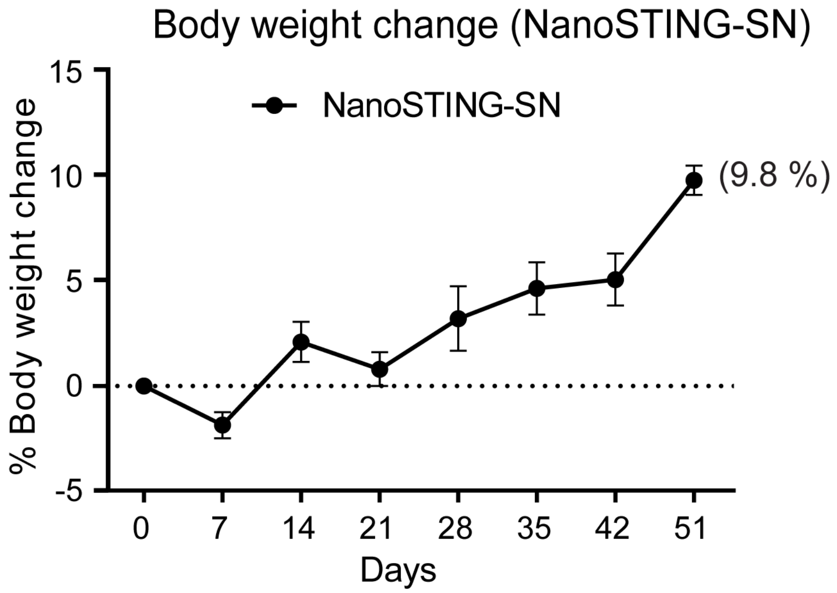

Supplementary Fig. 7: Percentage of bodyweight change of NanoSTING-SN vaccinated mice compared to the baseline (related to Figure 3).

Lines depict group mean bodyweight change from day 0; error bars represent SEM. Data presented as combined results from one independent experiment. Gender was not tested as a variable, and only female mice were used for the study.

Number of animals used: n=5/group

Source data are provided as a Source Data file.

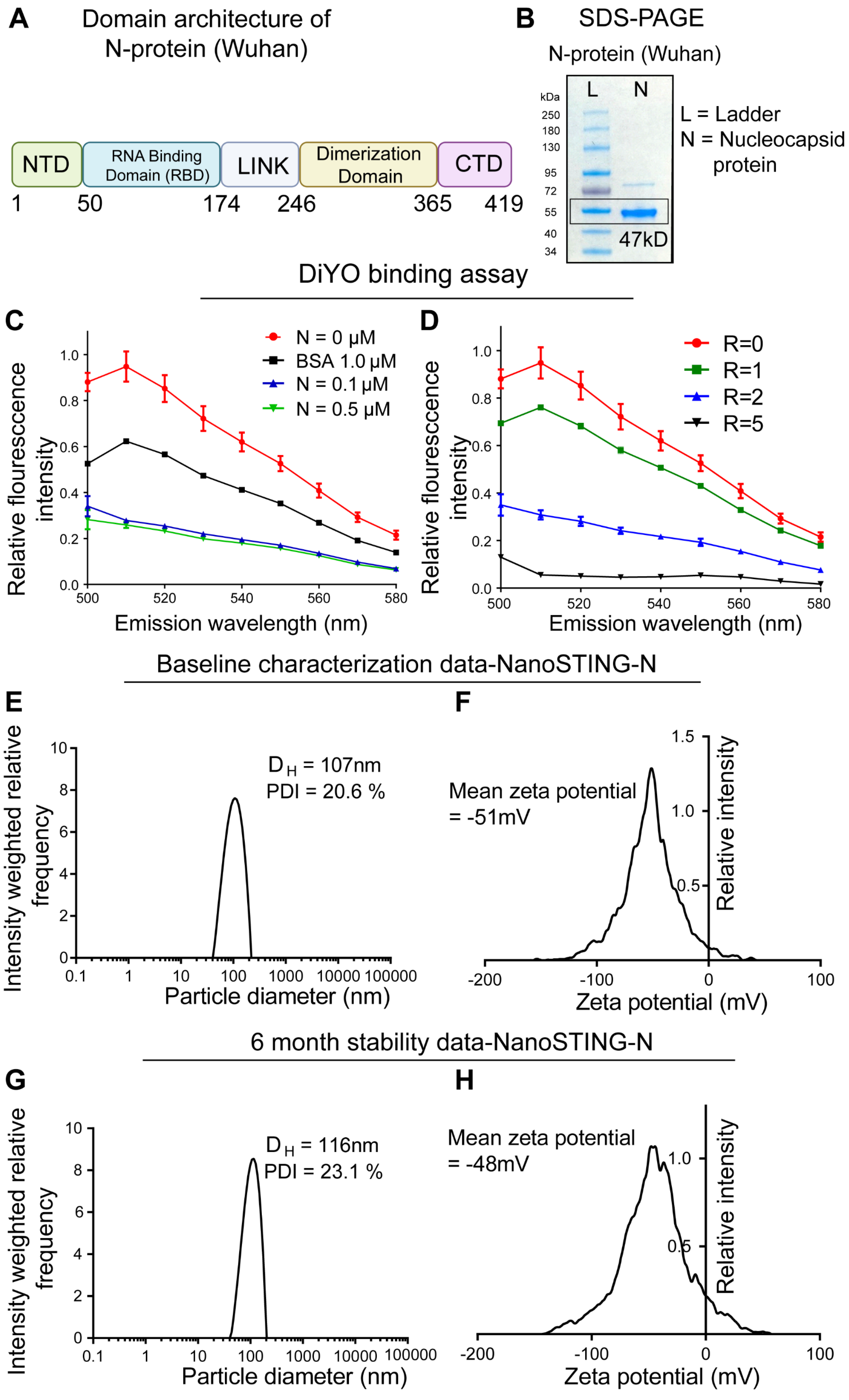
A
Domain architecture of N-protein (Wuhan)
NTD
RNA Binding Domain (RBD)
LINK
Dimerization Domain
CTD
1
50
174
246
365
419
B
SDS-PAGE
N-protein (Wuhan)
L
N
kDa
250
180
130
95
72
55
40
34
47kD
L = Ladder
N = Nucleocapsid protein
DiYO binding assay
C
N = 0 μM
BSA 1.0 μM
N = 0.1 μM
N = 0.5 μM
Relative flourescence intensity
Emission wavelength (nm)
D
R=0
R=1
R=2
R=5
Relative flourescence intensity
Emission wavelength (nm)
Baseline characterization data-NanoSTING-N
E
D H = 107nm
PDI = 20.6 %
Intensity weighted relative frequency
Particle diameter (nm)
F
Mean zeta potential = -51mV
Relative intensity
Zeta potential (mV)
6 month stability data-NanoSTING-N
G
D H = 116nm
PDI = 23.1 %
Intensity weighted relative frequency
Particle diameter (nm)
H
Mean zeta potential = -48mV
Relative intensity
Zeta potential (mV)

Supplementary Fig. 8: Characterization of NanoSTING-N (related to Figure 4).

(A) Domain structure of the SARS-CoV-2 N protein. **(B)** Denaturing SDS-PAGE gel of the purified nucleocapsid protein. **(C)** Fluorescence emission spectra of DNA-bound DiYO-1 in the presence of nucleocapsid-protein. Bovine serum albumin (BSA) was used as a control. **(D)** Fluorescence emission spectra of DNA-bound DiYO-1 in the presence of PEI (Positive control). **(E)** Distribution of NanoSTING-N particle sizes measured by DLS. **(F)** Zeta potential of the NanoSTING-N measured by ELS. **(G)** Distribution of NanoSTING-N particle sizes measured by DLS after storage at 4 °C for 6 months. **(H)** Zeta potential of the NanoSTING-N was measured by ELS after storage at 4 °C for 6 months. Abbreviations: NTD: N-terminal domain; CTD: C-terminal domain.

Source data are provided as a Source Data file.

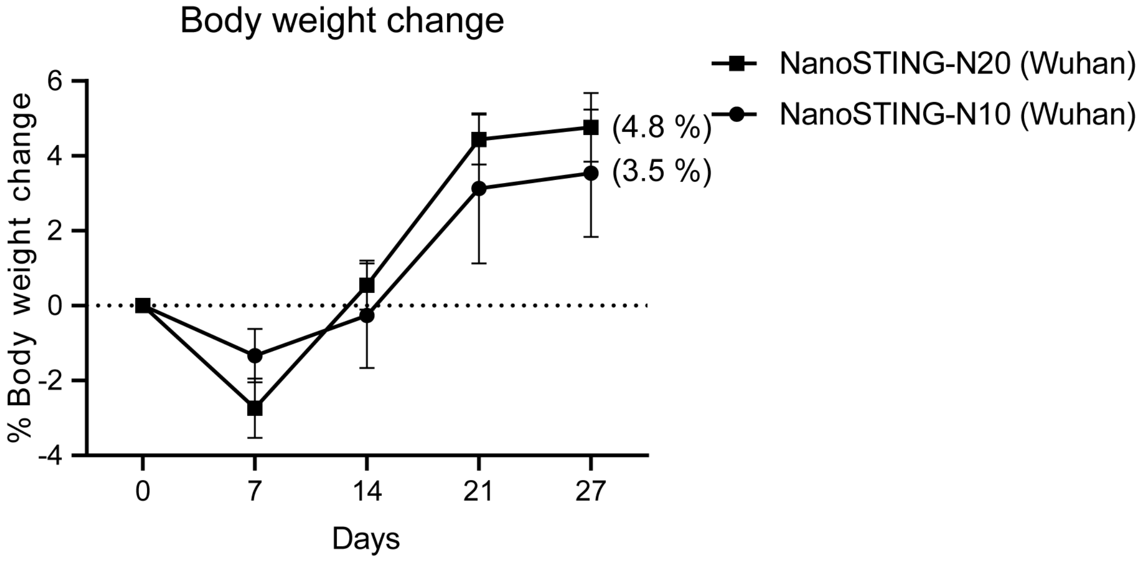

Supplementary Fig. 9: Percentage of bodyweight change of NanoSTING-N vaccinated mice compared to the baseline (related to Figure 4).

Lines depict group mean bodyweight change from day 0; error bars represent SEM. Data presented as combined results from one independent experiment. Gender was not tested as a variable for the study, and only female mice were included. Abbreviations: NanoSTING-N10: NanoSTING with 10 µg of Nucleocapsid protein, NanoSTING-N20: NanoSTING with 20 µg of Nucleocapsid protein

Number of animals used: n=4-5/group

Source data are provided as a Source Data file.

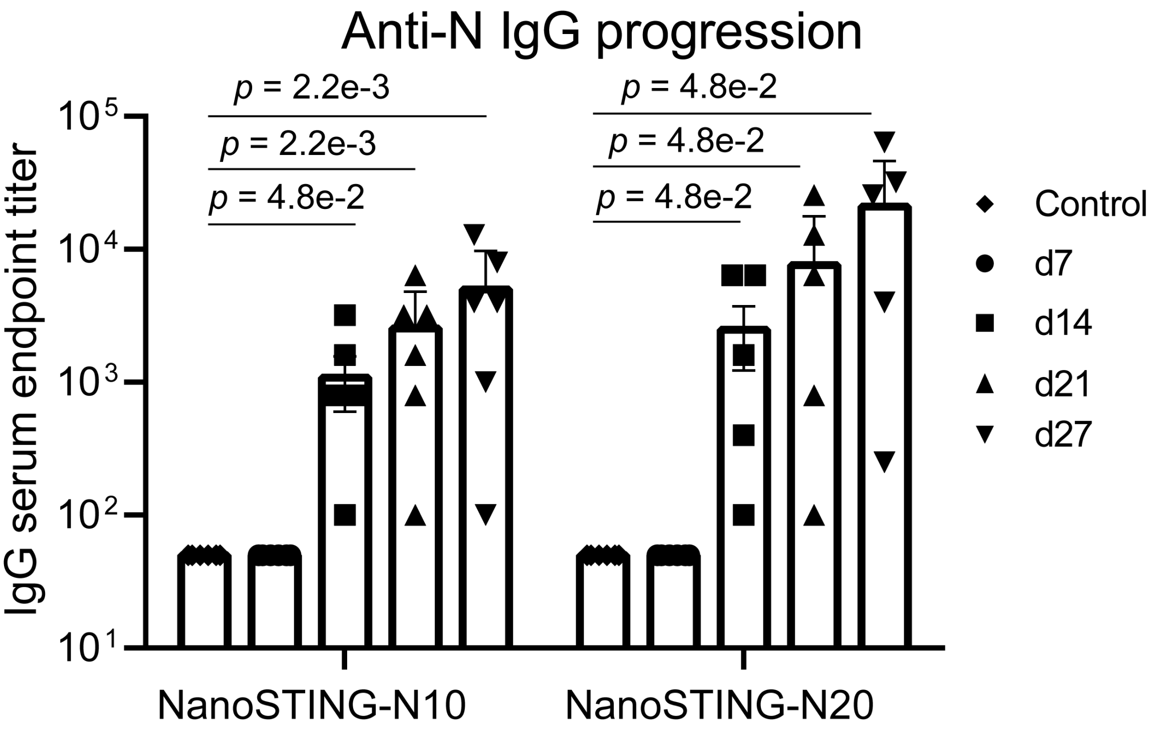

Supplementary Fig. 10: Anti-N IgG progression in the serum for NanoSTING-N10 and NanoSTING-N20 vaccinated mice at day 7, 14, 21, and 27 (related to Figure 4).

Individual data points represent biological replicates from separate animals; vertical bars show mean values with error bars representing SEM. Two-tailed mann-whitney U-test: *****p* < 0.0001; ****p* < 0.001; ***p* < 0.01; **p* < 0.05; ns: not significant. Data presented as combined results from one independent experiment. Gender was not tested as a variable, and only female mice were used for the study. Abbreviations: d7: Day 7, d14: Day 14, d21: Day 21, d27: Day 27.

Number of animals used: n=4-5/group

Source data are provided as a Source Data file.

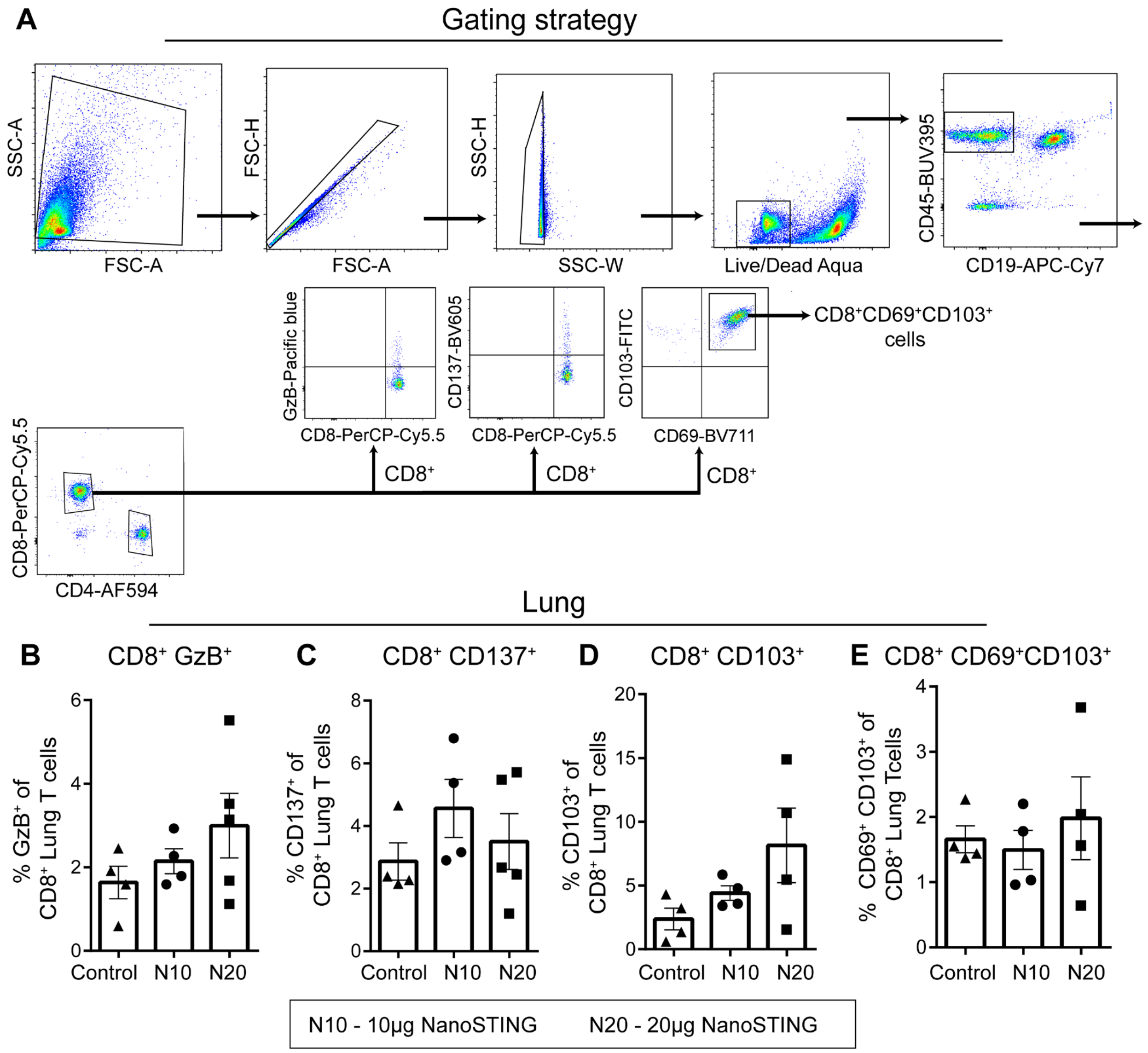

Supplementary Fig. 11: Lung CD8⁺ T cells were assayed for expression of Granzyme B (GrB), CD137, CD103, and CD69 by flow cytometry (related to Figure 4).

(A) Flow cytometric gating strategy for the identification and quantification of N protein reactive CD8⁺ T cells: FSC-A vs FSC-H and SSC-H vs SSC-W parameters were used to exclude doublets. In addition, the live cell population was selected as Live/Dead Aqua negative cells. From the live cells, we gated on CD45⁺ cells in both lung and spleen samples. Within the CD45⁺ cells, we identified CD4⁺ and CD8⁺ subsets. Next, we gated on the CD8⁺ cells and identified specific subpopulations, including cytotoxic T cells (CD8⁺ GzB⁺), activated T cells (CD8⁺ CD137⁺), and lung resident memory T cells (CD8⁺ CD103⁺, and CD8⁺ CD103⁺ CD69⁺ cells). **(B-E)** Lung cells were stimulated *ex vivo* with overlapping peptide pools, and the expression of GzB⁺ (B), CD137⁺ (C), CD103⁺ (D), CD103⁺ and CD69⁺

(E) expression in $CD8^+$ T cells were quantified using flow cytometry. Individual data points represent biological replicates from separate animals; vertical bars show mean values with error bar representing SEM. Two-tailed mann-whitney U-test: ****$p < 0.0001$; ***$p < 0.001$; **$p < 0.01$; *$p < 0.05$; ns: not significant. Gender was not tested as a variable, and only female mice were used for the study. Data presented as combined results from one independent experiment. Abbreviations: SSC-A: side scatter area, FSC-A: forward scatter area, FSC-H: forward scatter height, SSC-H: side scatter height, SSC-W: side scatter width.

Number of animals used: n=4-5/group

Source data are provided as a Source Data file.

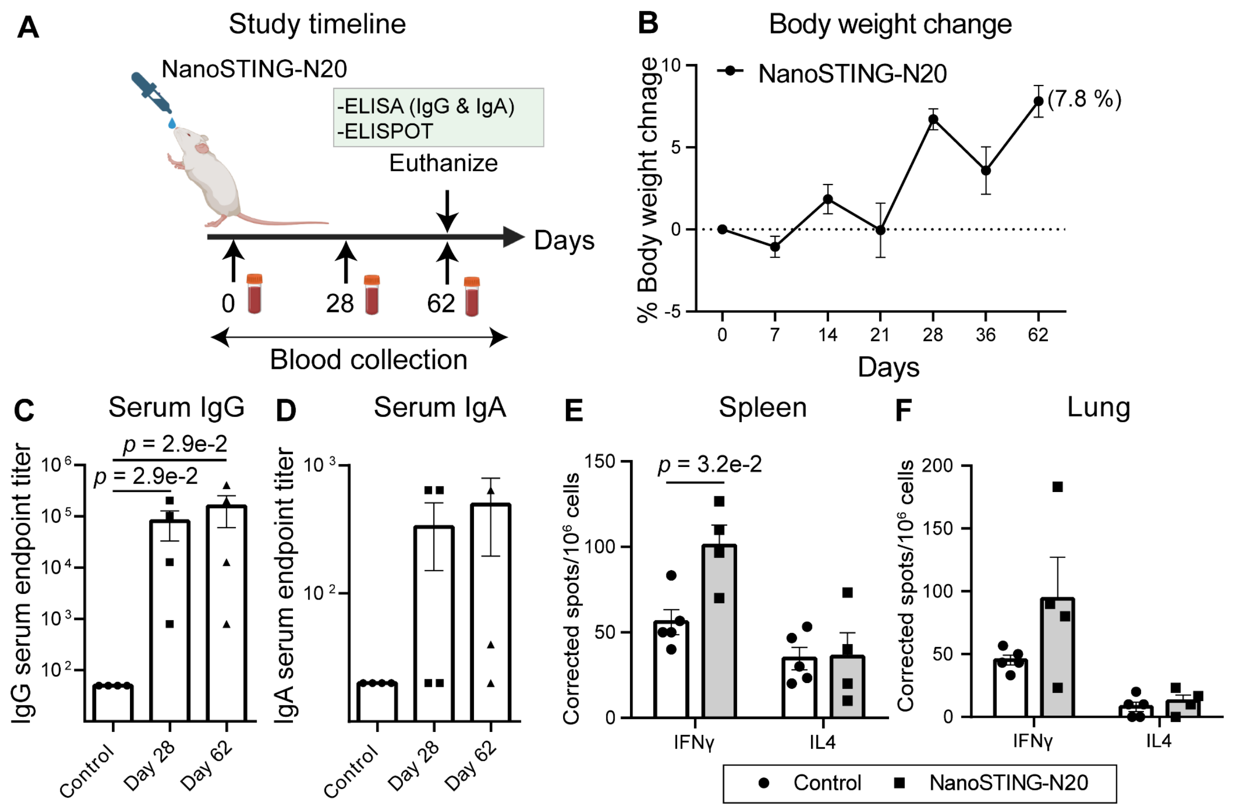

Supplementary Fig. 12: Immunization with NanoSTING-N elicits strong cellular and humoral immune responses

(**A**) Experimental set up: We immunized *BALB/c* mice (n=4/group) intranasally with single dose of NanoSTING-N followed by the collection of serum after day 28 and day 62. The body weight of the animals were monitored every week after the immunization. We euthanized the animals at day 62 followed by the collection of serum, lungs, and spleen. Bodyweight change, ELISA (IgG & IgA), and ELISPOT (IFNγ and IL4) were used as primary endpoints. Naïve *BALB*/c mice were used controls (n=4-5/group). (**B**) Percent bodyweight change compared to the baseline at the indicated time intervals. (**C, D**) Humoral immune responses in the serum were evaluated using N-protein based IgG and IgA ELISA. (**E, F**) Splenocytes (**E**) and lung cells (**F**) were stimulated ex vivo with overlapping N and S-peptide pools, and IFNγ & IL4 responses were detected by ELISPOT. Individual data points represent independent biological replicates taken from separate animals; vertical bars show mean values with error bar representing SEM. Each dot represents an individual mouse. For C-F, the analysis was performed using two-tailed Mann-Whitney U-test: ****$p < 0.0001$; ***$p < 0.001$; **$p < 0.01$; *$p < 0.05$; ns: not significant. Data presented as combined results from one independent experiment. Gender was not tested as a variable, and only female mice were used for the study. Supp. Fig. 12A Created with BioRender.com released under a Creative Commons Attribution-NonCommercial-NoDerivs 4.0 International license (https://creativecommons.org/licenses/by-nc-nd/4.0/deed.en).

Number of animals used: n=4/group

Source data are provided as a Source Data file.

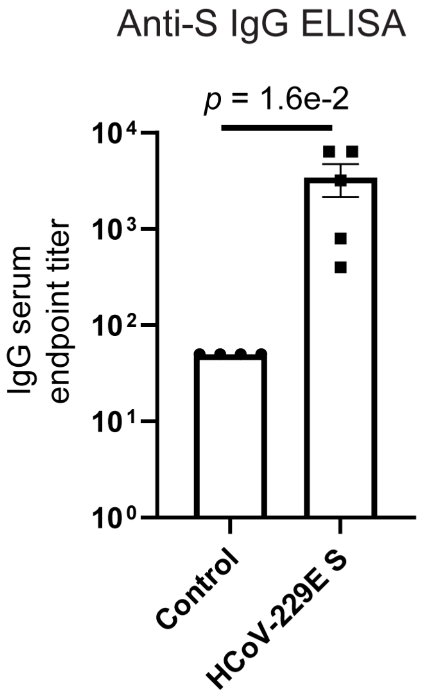

Supplementary Fig. 13: Humoral immune responses in the serum of NanoSTING-NS vaccinated animals were evaluated using an S protein-based IgG ELISA (related to Figure 7).

Individual data points represent independent biological replicates taken from separate animals; vertical bars show mean values with error bars representing SEM. Each dot represents an individual mouse. The analysis was performed using two-tailed Mann-Whitney U-test: ****p < 0.0001; ***p < 0.001; **p < 0.01; *p < 0.05; ns: not significant. Data presented as combined results from one independent experiment. Gender was not tested as variable for the study, and only female mice were used. Abbreviations: HCoV-229E S: human coronavirus 229E spike protein.

Number of animals used: n=4-5/group

Source data are provided as a Source Data file.

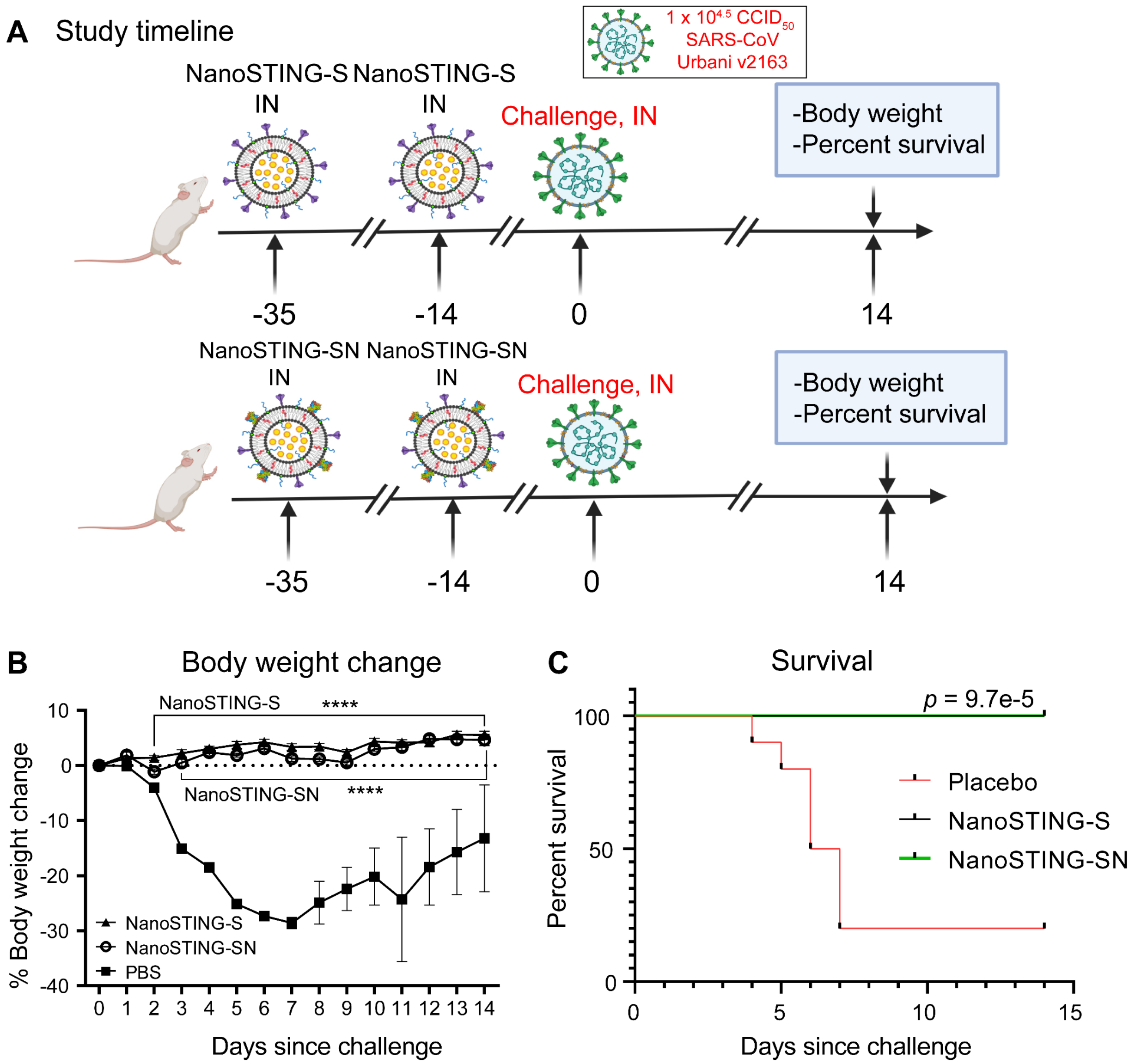

Supplementary Fig. 14: Dual-dose immunization of mice with NanoSTING-S & NanoSTING-SN vaccine yields cross-reactive humoral immunity against alpha coronaviruses and confers protection against SARS-CoV (related to Figure 8)

(A) Experimental set up for SARS-CoV challenge studies in mice. We immunized mice (n=10/group) intranasally with one dose of either NanoSTING-S or NanoSTING-SN on day -35 and a second dose on day -14 and challenged the mice intranasally with the SARS-CoV (v2163 strain) on day 0. Post-challenge, we monitored the animals for 14 days for changes in body weight and survival. **(B)** Percent body weight change of mice compared to the baseline at the indicated time intervals. **(C)** Percent survival of mice compared to the baseline at the indicated time intervals. Individual data points represent independent biological replicates taken from separate animals; vertical bars show mean values with error bars representing SEM. Each dot represents an individual mouse. For B,

the data was compared via a mixed-effects model for repeated measures analysis. Lines depict group mean bodyweight change from day 0; error bars represent SEM. For B, the exact p-values comparing the NanoSTING-S/NanoSTING-SN group to the PBS group are Day 2: p = 1.1e-6/ns, Day 3: p = 1.1e-13/1.1e-13, Day 4: p = 1.1e-13/1.1e-13, Day 5: p =1.1e-13/1.1e-13, Day 6: p = 1.1e-13/1.1e-13, Day 7: p =1.1e-13/1.1e-13, Day 8: p =1.1e-13/1.1e-13, Day 9: p =1.1e-13/1.1e-13, Day 10: p =1.1e-13/1.1e-13, Day 11: p =1.1e-13/1.1e-13, Day 12: p =1.1e-13/1.1e-13, Day 13: p =1.1e-13/1.3e-13 and Day 14: 1.3e-13/1.4e-13. Asterisks indicate significance compared to the PBS-treated animals at each time point. Data presented as combined results from one independent experiment. Gender was tested as a variable with an equal number of male and female mice included in the study. Figure 14A, I Created with BioRender.com released under a Creative Commons Attribution-NonCommercial-NoDerivs 4.0 International license (https://creativecommons.org/licenses/by-nc-nd/4.0/deed.en)

Number of animals used: n=5-10/group

Source data are provided as a Source Data file.

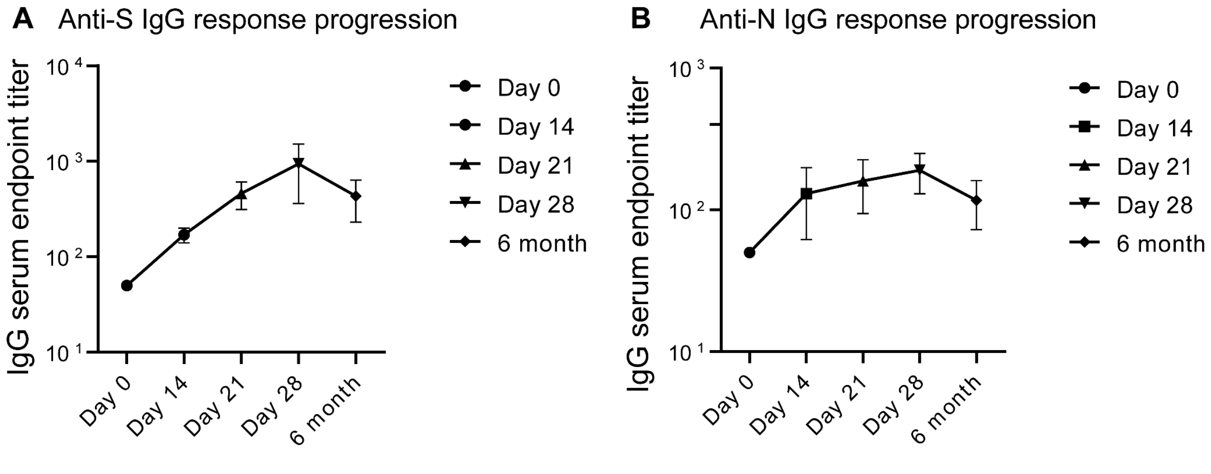

Supplementary Fig. 15: Durability of the IgG responses in rhesus macaques (related to Figure 8)

We measured the serum IgG titers in rhesus macaques post-immunization with NanoSTING-SN. **(A)** Anti-S IgG response progression at indicated time points. **(B)** Anti-N IgG response progression at indicated time points. Lines depict group mean IgG serum end point titers for indicated time points; error bars represent SEM. Data presented as combined results from one independent experiment. Two male NHPs and three female NHP were taken for the study.

Number of animals used: n=5/group

Source data are provided as a Source Data file.

SUPPLEMENTARY METHODS

To quantify the kinetics of SARS-CoV-2 infection in the upper respiratory tract (URT) upon different antigens used for immunization [Spike(S), Nucleocapsid(N) and N+S], we modified the innate immune model as described previously[46]. If S immunization works by *de novo* blocking of viral entry through neutralizing antibodies and N immunization works by inducing cytotoxic T cell responses, we modified the governing equations as shown in the **Supplementary Table1** below and **Figure 2** of the main manuscript.

No immunization	S immunization	N immunization	N+S immunization
$\frac{d\mathbf{T}}{dt} = -\beta V\mathbf{T} - \varphi I\mathbf{T} + \rho\mathbf{R}$<br>$\frac{d\mathbf{R}}{dt} = \varphi I\mathbf{T} - \rho\mathbf{R}$<br>$\frac{d\mathbf{E}}{dt} = \beta V\mathbf{T} - k\mathbf{E}$<br>$\frac{d\mathbf{I}}{dt} = k\mathbf{E} - \sigma\mathbf{I}$<br>$\frac{d\mathbf{V}}{dt} = \pi\mathbf{I} - c\mathbf{V}$	$\frac{d\mathbf{T}}{dt} = -\beta(1-g)V\mathbf{T} - (\varphi\mathbf{I})\mathbf{T} + \rho\mathbf{R}$<br>$\frac{d\mathbf{R}}{dt} = (\varphi\mathbf{I})\mathbf{T} - \rho\mathbf{R}$<br>$\frac{d\mathbf{E}}{dt} = \beta(1-g)V\mathbf{T} - k\mathbf{E}$<br>$\frac{d\mathbf{I}}{dt} = k\mathbf{E} - \sigma\mathbf{I}$<br>$\frac{d\mathbf{V}}{dt} = \pi\mathbf{I} - c\mathbf{V}$	$\frac{d\mathbf{T}}{dt} = -\beta V\mathbf{T} - \varphi I\mathbf{T} + \rho\mathbf{R}$<br>$\frac{d\mathbf{R}}{dt} = \varphi I\mathbf{T} - \rho\mathbf{R}$<br>$\frac{d\mathbf{E}}{dt} = \beta V\mathbf{T} - k\mathbf{E}$<br>$\frac{d\mathbf{I}}{dt} = k\mathbf{E} - (\sigma + w)\mathbf{I}$<br>$\frac{d\mathbf{V}}{dt} = \pi\mathbf{I} - c\mathbf{V}$	$\frac{d\mathbf{T}}{dt} = -\beta(1-g)V\mathbf{T} - (\varphi\mathbf{I})\mathbf{T} + \rho\mathbf{R}$<br>$\frac{d\mathbf{R}}{dt} = (\varphi\mathbf{I})\mathbf{T} - \rho\mathbf{R}$<br>$\frac{d\mathbf{E}}{dt} = \beta(1-g)V\mathbf{T} - k\mathbf{E}$<br>$\frac{d\mathbf{I}}{dt} = k\mathbf{E} - (\sigma + w)\mathbf{I}$<br>$\frac{d\mathbf{V}}{dt} = \pi\mathbf{I} - c\mathbf{V}$

Supplementary Table 1: Equations governing anti-viral response for different modes of immunizations. Abbreviations: T = Target cells, R = Refractory cells, E = Eclipse phase cell (Infected cells not producing virus), I = Infected cells productively making virus, V = Viral load

We solved these ordinary differential equations with mean population parameter values and initial values taken from Ke et al and as shown in **Supplementary Table 2 & 3**. We calculated the Viral load area under the curve (AUC) during infection for varying de-novo blocking efficiency (□) and cytotoxic killing efficacy (□).

Parameter	Description	Mean population value/unit
β	Infectivity parameter constant	$3.2*10^{-8}$ ml/RNA copy/day
σ	Death rate of infected cells	1.7 /day
π	Composite parameter for virus production and sampling	45.3/ml/day
φ	Rate constant for interferon induced conversion of Target cells to refractory cells	$1.3*10^{-6}$ /cell/day
k	1/the eclipse phase duration	4 /day

c	virus clearance rate	10 /day
ρ	Rate at which refractory cells become target cells again	0.0044/day
□	De-novo blocking efficacy<br>(Antibody efficiency in blocking viral entry)	0-1 (Variable)
□	Death rate of infected cells mediated by cytotoxic T cells	Variable /day

Supplementary Table 2: Mean population values of parameters for equations in Supplementary Table 1.

Variable	Initial value
T0 – Total number of target cells	$8*10^7$
E0 – Initial number of Infected cells	5, 500

Supplementary Table 3: Initial values for total number of target cells(T0) and initial number of infected cells(I0) upon viral exposure.

Antibody/Conjugate	Color	Clone; Company; Catalog number	Dilution
CD4	AF594	GK1.5; Biolegend #100446	1:100
CD8b	PerCP-Cy5.5	GK1.5; Biolegend #100446,	1:200
CD69	BV711	H1.2F3; Biolegend #104537	1:100
CD137	BV605	1AH2; BD #740364	1:100
CD45	BUV395	30-F11; BD #564279	1:200
IFNγ	AF488	XMG1.2; BD #557735	1:200
Granzyme B	Pacific Blue	GB11; Biolegend #515407	1:300
CD103	FITC	2E7; Biolegend #121420	1:100
CD19	APC-Cy7	6D5; Biolegend #115529	1:200

Supplementary Table 4: List of antibodies used for flow cytometry

Sup Note 1

```
% Covid dynamics with NanoSTING
%beta - Infectivity parameter constant = 3.2*10^-8 ml/RNA copy/day
%delta - Death rate of infected cells - 1.7/day
%pii - Composite parameter for virus production and sampling - 45.3/ml/day
%phi - Rate constant for interferon induced conversion of Target cells to
%refractory cells - 1.3*10^-6 /cell/day
%rho - Rate at which refractory cells become target cells again -0.0044/day
%c - Virus clearance rate - 10/day
%k - 1/the eclipse phase duration = 4/day

beta  = 3.2*10^-8; %ml/RNA copy/day
delta = 1.7; %/day
pii = 45.3; %/ml/day
phi = 1.3*10^-6; %/cell/day
rho = 0.0044; %/day
c   = 10; %/day
k   = 4; %/day
denovo = 0.8; % coefficient responsible for denovo-blocking
cyT = 0.6; % % killing rate of cytotoxic T cells

T0 = 8*10^7; %Total number of target cells
R0 = 0; %Initial refractory cells
E0 = 5; %Initial number of infected cells
I0 = 0;
V0 = 0; %Initial virus titer
```

```
t_int = [0,30];
init_cond = [T0,R0,E0,I0,V0]';
[t,y] = ode45(@(t,Y) covidode(t,Y,beta,delta,pii,phi,rho,c,k,denovo,cyT), t_int,init_cond );
```

Function handle:

```
function dYdt = covidode(t,Y,beta,delta,pii,phi,rho,c,k,denovo,cyT )
dYdt = [ -beta*(1-denovo)*Y(5)*Y(1)-(phi*Y(4))*Y(1)+rho*Y(2);
        (phi*Y(4))*Y(1)-rho*Y(2);
        beta*(1-denovo)*Y(5)*Y(1)-k*Y(3);
        k*Y(3)-delta*Y(4)-cyT*Y(4);
        pii*Y(4)-c*Y(5)];
end
```
