# Supplementary material for: Multi-antigen intranasal vaccine protects against challenge with sarbecoviruses and prevents transmission in hamsters
Source: Nat Commun. 2024 Jul 23;15:6193. doi: 10.1038/s41467-024-50133-2 (PMC11266618; doi:10.1038/s41467-024-50133-2)
Supplement: Supplementary file 3 — Reporting Summary [file 41467_2024_50133_MOESM3_ESM.pdf]

nature portfolio

Corresponding author(s): Navin Varadarajan

Last updated by author(s): Jun 9, 2024

Reporting Summary

Nature Portfolio wishes to improve the reproducibility of the work that we publish. This form provides structure for consistency and transparency in reporting. For further information on Nature Portfolio policies, see our Editorial Policies and the Editorial Policy Checklist.

Statistics

For all statistical analyses, confirm that the following items are present in the figure legend, table legend, main text, or Methods section.

n/a	Confirmed	
☐	☒	The exact sample size (*n*) for each experimental group/condition, given as a discrete number and unit of measurement
☐	☒	A statement on whether measurements were taken from distinct samples or whether the same sample was measured repeatedly
☐	☒	The statistical test(s) used AND whether they are one- or two-sided<br>*Only common tests should be described solely by name; describe more complex techniques in the Methods section.*
☒	☐	A description of all covariates tested
☒	☐	A description of any assumptions or corrections, such as tests of normality and adjustment for multiple comparisons
☐	☒	A full description of the statistical parameters including central tendency (e.g. means) or other basic estimates (e.g. regression coefficient) AND variation (e.g. standard deviation) or associated estimates of uncertainty (e.g. confidence intervals)
☐	☒	For null hypothesis testing, the test statistic (e.g. *F*, *t*, *r*) with confidence intervals, effect sizes, degrees of freedom and *P* value noted<br>*Give P values as exact values whenever suitable.*
☒	☐	For Bayesian analysis, information on the choice of priors and Markov chain Monte Carlo settings
☒	☐	For hierarchical and complex designs, identification of the appropriate level for tests and full reporting of outcomes
☒	☐	Estimates of effect sizes (e.g. Cohen's *d*, Pearson's *r*), indicating how they were calculated

Our web collection on statistics for biologists contains articles on many of the points above.

Software and code

Policy information about availability of computer code

Data collection	Characterization of NanoSTING-S, NanoSTING-N and NanoSTING-NS was performed using DLS and zeta sizer on Litesizer 500 (Anton Paar) in automatic mode. THP-1 dual assay, ELISA and ELISPOT data was collected using Cytation 7, Bio-Tek Instruments, Inc., Gen5 Software (Version 3.10.06). Flow cytometry data was collected on LSR-Fortessa flow cytometer (BD Bioscience).
Data analysis	The statistical analyses were performed in the Prism (GraphPad) software package (version v6.07). Parts of the figures were made in BioRendor and Figures were assembled using Adobe Illustrator (version CS6). Flow cytometry data was analyzed using using FlowJo™ software version 10.8 (Tree Star Inc, Ashland, OR, USA). For quantitative modelling, we solved the system of ordinary differential equations (ODEs) for different S and N response efficiencies using the ODE45 function in MATLAB 2018b. A sample MATLAB code for solving the system of equations has been provided in Sup note 1.

For manuscripts utilizing custom algorithms or software that are central to the research but not yet described in published literature, software must be made available to editors and reviewers. We strongly encourage code deposition in a community repository (e.g. GitHub). See the Nature Portfolio guidelines for submitting code & software for further information.

Data

Policy information about availability of data

All manuscripts must include a data availability statement. This statement should provide the following information, where applicable:

- Accession codes, unique identifiers, or web links for publicly available datasets
- A description of any restrictions on data availability
- For clinical datasets or third party data, please ensure that the statement adheres to our policy

All data are included in the source data file or available from the authors, as are unique reagents used in this article. The raw numbers for charts and graphs are available in the Source Data file whenever possible. All material and experimental data requests should be directed to the corresponding author, Navin Varadarajan. Source data are provided with this paper.

Research involving human participants, their data, or biological material

Policy information about studies with human participants or human data. See also policy information about sex, gender (identity/presentation), and sexual orientation and race, ethnicity and racism.

Reporting on sex and gender	No humans were used in this study.
Reporting on race, ethnicity, or other socially relevant groupings	*Please specify the socially constructed or socially relevant categorization variable(s) used in your manuscript and explain why they were used. Please note that such variables should not be used as proxies for other socially constructed/relevant variables (for example, race or ethnicity should not be used as a proxy for socioeconomic status).*<br>*Provide clear definitions of the relevant terms used, how they were provided (by the participants/respondents, the researchers, or third parties), and the method(s) used to classify people into the different categories (e.g. self-report, census or administrative data, social media data, etc.)*<br>*Please provide details about how you controlled for confounding variables in your analyses.*
Population characteristics	*Describe the covariate-relevant population characteristics of the human research participants (e.g. age, genotypic information, past and current diagnosis and treatment categories). If you filled out the behavioural & social sciences study design questions and have nothing to add here, write "See above."*
Recruitment	*Describe how participants were recruited. Outline any potential self-selection bias or other biases that may be present and how these are likely to impact results.*
Ethics oversight	*Identify the organization(s) that approved the study protocol.*

Note that full information on the approval of the study protocol must also be provided in the manuscript.

Field-specific reporting

Please select the one below that is the best fit for your research. If you are not sure, read the appropriate sections before making your selection.

☒ Life sciences ☐ Behavioural & social sciences ☐ Ecological, evolutionary & environmental sciences

For a reference copy of the document with all sections, see nature.com/documents/nr-reporting-summary-flat.pdf

Life sciences study design

All studies must disclose on these points even when the disclosure is negative.

Sample size	Sample size was determined based on similar studies in this field.
Data exclusions	No data was excluded from the analyses.
Replication	Animal studies were performed in biological triplicates or more as indicated in the figure legends. When applicable, technical repeats is specified for each experiment in the figure legends. Reproducibility between animals in NanoSTING, NanoSTING-N and NanoSTING-NS, Oseltamivir and naïve controls/PBS treated groups is shown in the results.
Randomization	Animals were randomly divided into experimental groups.
Blinding	The pathologists performing the histopathological analysis were blinded to treatment. The adjuvant was manufactured at UH and the adjuvant/protein were shipped to USU. USU performed the vaccine formulation for the challenge experiments, immunized and challenged the animals.

Reporting for specific materials, systems and methods

We require information from authors about some types of materials, experimental systems and methods used in many studies. Here, indicate whether each material, system or method listed is relevant to your study. If you are not sure if a list item applies to your research, read the appropriate section before selecting a response.

Materials & experimental systems

n/a	Involved in the study
☐	☒ Antibodies
☐	☒ Eukaryotic cell lines
☒	☐ Palaeontology and archaeology
☐	☒ Animals and other organisms
☒	☐ Clinical data
☒	☐ Dual use research of concern
☒	☐ Plants

Methods

n/a	Involved in the study
☒	☐ ChIP-seq
☐	☒ Flow cytometry
☒	☐ MRI-based neuroimaging

Antibodies

Antibodies used

1. anti-CD4 AF589 (clone GK1.5; Biolegend #100446, Lot no B297643)
2. anti-CD8b (clone YTS156.7.7; Biolegend #126609, Lot no. B304118)
3. anti-CD69 (clone H1.2F3; Biolegend #104537, Lot no B319210)
4. anti-CD137 (clone 1AH2; BD; # 740364, Lot no. 1039861)
5. anti-CD45 (clone 30-F11; BD; #564279, Lot no 0022143)
6. Interferon (IFN) gamma (clone XMG1.2; BD; #557735, Lot no. B363654)
7. anti-Granzyme B (clone GB11; Biolegend; #515407, Lot no. 515407)
8. anti-CD103 (Clone 2E7; Biolegend #121420)

Validation

All antibodies are commercially available and validated by manufacturers. Additionally information can be found on product website, listed below.
1. https://www.biolegend.com/en-us/products/alexa-fluor-594-anti-mouse-cd4-antibody-9412
2. https://www.biolegend.com/en-us/products/percp-cyanine5-5-anti-mouse-cd8b-antibody-4477
3. https://www.biolegend.com/en-us/products/brilliant-violet-711-anti-mouse-cd69-antibody-12139
4. https://www.bdbiosciences.com/en-us/products/reagents/flow-cytometry-reagents/research-reagents/single-color-antibodies-ruo/bv605-rat-anti-mouse-cd137.740364
5. https://www.bdbiosciences.com/en-us/products/reagents/flow-cytometry-reagents/research-reagents/single-color-antibodies-ruo/buv395-rat-anti-mouse-cd45.564279
6. https://www.bdbiosciences.com/en-us/products/reagents/flow-cytometry-reagents/research-reagents/single-color-antibodies-ruo/alexa-fluor-647-rat-anti-mouse-ifn.557735
7. https://www.biolegend.com/en-us/products/pacific-blue-anti-human-mouse-granzyme-b-antibody-8612
8. https://www.biolegend.com/en-ie/products/fitc-anti-mouse-cd103-antibody-7053?GroupID=BLG4646

Eukaryotic cell lines

Policy information about cell lines and Sex and Gender in Research

Cell line source(s)

NF-κB-SEAP IRF-Luc Reporter Monocytes- THP1-Dual™ Cells (human, Invivogen: Cat No. thpd-nfis)
African green monkey kidney cells-VeroE6 (ATCC®, cat# CRL-1586'M)
Madin-Darby canine kidney- MDCK cells (ATCC®, cat# CCL-34)

Authentication

All cell lines were purchased directly from Invitrogen and ATCC.

Mycoplasma contamination

The cell lines were tested negative for mycoplasma.

Commonly misidentified lines
(See ICLAC register)

No commonly misidentified cell lines were used in this study.

Animals and other research organisms

Policy information about studies involving animals; ARRIVE guidelines recommended for reporting animal research, and Sex and Gender in Research

Laboratory animals

In this study were used BALB/c mice (9-10 weeks old) and Syrian Golden hamsters (Mesocricetus auratus) median age 6 to 10 weeks old. We purchased the animals from Jackson Laboratory and Charles River. Rhesus macaques (Macaca mulatta; Indian origin, between 4 and 11 years of age and 4 – 12 kg in weight) were used.

Wild animals

No wild animals were used in this study.

Reporting on sex

For all the viral challenge studies done at UH and USU, equal number of male and female mice and hamsters were used for the study. For non-human primates study, we used 2 females and one male.

Field-collected samples

No field-collected samples were used in this study.

Ethics oversight

The mouse, hamster and NHP studies were performed under the study protocol (PROTO2020000019, PROTO202100006, PROTO202100049, PROTO202200025), as approved by the Institutional Animal Care and Use Committee in University of Houston. The animal experiments at USU were conducted in accordance with an approved protocol by the Institutional Animal Care and Use Committee of Utah State University. The work was performed in the AAALAC-accredited LARC of the university in accordance with the National Institutes of Health Guide for the Care and Use of Laboratory Animals (8th edition; 2011).

Note that full information on the approval of the study protocol must also be provided in the manuscript.

Flow Cytometry

Plots

Confirm that:

- [x] The axis labels state the marker and fluorochrome used (e.g. CD4-FITC).
- [ ] The axis scales are clearly visible. Include numbers along axes only for bottom left plot of group (a 'group' is an analysis of identical markers).
- [x] All plots are contour plots with outliers or pseudocolor plots.
- [x] A numerical value for number of cells or percentage (with statistics) is provided.

Methodology

Sample preparation

As described in the methods section of the paper, we isolated the lung cells from minced tissues and the splenocytes from the homogenized spleen of the mice. We stimulated the spleen and lung cells from immunized and control animals to detect nucleocapsid protein-specific CD8+ T cell responses with an N protein-peptide pool at a concentration of 1.5 µg/mL/peptide (Miltenyi Biotec; 130-126-699, Germany) at 37 °C for 16-18 h followed by the addition of Brefeldin A (5 µg/ml BD Biosciences #BD 555029) for the last 5 h of the incubation. We used 10 ng/ml PMA (Sigma, St. Louis, MI, USA) and 1 µg/ml ionomycin (Sigma, St. Louis, MI, USA) as the positive control. Stimulation without the peptides served as background control. We collected the cells and stained with Live/Dead Aqua (Thermo Fisher #L34965) in PBS, followed by Fc-receptor blockade with anti-CD16/CD32 (Thermo Fisher #14-0161-85), and then stained for 30 min on ice with the following antibodies in flow cytometry staining buffer (FACS): anti-CD4 AF589 (clone GK1.5; Biolegend #100446), anti-CD8b (clone YTS156.7.7; Biolegend #126609), anti-CD69 (clone H1.2F3; Biolegend #104537), anti-CD137 (clone 1AH2; BD; # 40364), anti-CD45 (clone 30-F11; BD; #564279). We washed the cells twice with the FACS buffer. We then fixed them with 100 µL IC (intracellular) fixation buffer (eBioscience) for 30 min at RT. We permeabilized the cells for 10 min with 200 µL permeabilization buffer (BD Cytofix solution kit). We performed the intracellular staining using the antibodies Alexa Fluor 488 interferon (IFN) gamma (clone XMG1.2; BD; #557735) and Granzyme B (clone GB11; Biolegend; #515407) overnight at 4 °C. Next, we washed the cells with FACS buffer and analyzed them on LSR-Fortessa flow cytometer (BD Bioscience) using FlowJo™ software version 10.8 (Tree Star Inc, Ashland, OR, USA). We calculated the results as the total number of cytokine-positive cells with background subtracted. We optimized the amount of the antibodies by titration

Instrument

LSR-Fortessa flow cytometer (BD Bioscience)

Software

FlowJo™ software version 10.8 (Tree Star Inc, Ashland, OR, USA)

Cell population abundance

N/A

Gating strategy

FSC-A vs FSC-H and SSC-H vs SSC-W parameters were used to exclude doublets. In addition, the live cell population was selected as Live/Dead Aqua negative cells. From the live cells, we gated on CD45+ cells in both lung and spleen samples. Within the CD45+ cells, we identified CD4+ and CD8+ subsets. Next, we gated on the CD8+ cells and identified specific subpopulations, including cytotoxic T cells (CD8+ GzB+), activated T cells (CD8+ CD137+) and lung resident memory T cells (CD8+ CD103+, and CD8+ CD103+ CD69+ cells).

- [x] Tick this box to confirm that a figure exemplifying the gating strategy is provided in the Supplementary Information.
